# Frail hypertensive older adults with prediabetes and chronic kidney disease: insights on organ damage and cognitive performance - preliminary results from the CARYATID study

**DOI:** 10.1186/s12933-024-02218-x

**Published:** 2024-04-10

**Authors:** Gaetano Santulli, Valeria Visco, Michele Ciccarelli, Mario Nicola Vittorio Ferrante, Piero De Masi, Antonella Pansini, Nicola Virtuoso, Armando Pirone, Germano Guerra, Veronica Verri, Gaetano Macina, Alessandro Taurino, Klara Komici, Pasquale Mone

**Affiliations:** 1 Department of Medicine, Division of Cardiology, Wilf Family Cardiovascular Research Institute, Einstein – Mount Sinai Diabetes Research Center (ES-DRC), Einstein Institute for Aging Research, Albert Einstein College of Medicine, New York, NY USA; 2 Department of Molecular Pharmacology, Einstein Institute for Neuroimmunology and Inflammation (INI), Fleischer Institute for Diabetes and Metabolism (FIDAM), Albert Einstein College of Medicine, New York, NY USA; 3 Department of Advanced Biomedical Sciences, University of Naples “Federico II”, Fisciano, Italy; 4 International Translational Research and Medical Education (ITME) Consortium, Academic Research Unit, Naples, Italy; 5 Department of Medicine, Surgery and Dentistry, University of Salerno, Baronissi, Italy; 6 ASL Avellino, Avellino, Italy; 7 Cardiology Unit, University Hospital “San Giovanni di Dio e Ruggi d’Aragona”, Salerno, Italy; 8 Department of Medicine and Health Sciences “Vincenzo Tiberio”, University of Molise, Campobasso, Italy; 9 University of Bari, Bari, Italy; 10 Casa di Cura “Montevergine”, Mercogliano, Avellino, Italy

## Abstract

**Background:**

Hypertension and chronic kidney disease (CKD) pose significant public health challenges, sharing intertwined pathophysiological mechanisms. Prediabetes is recognized as a precursor to diabetes and is often accompanied by cardiovascular comorbidities such as hypertension, elevating the risk of pre-frailty and frailty. Albuminuria is a hallmark of organ damage in hypertension amplifying the risk of pre-frailty, frailty, and cognitive decline in older adults. We explored the association between albuminuria and cognitive impairment in frail older adults with prediabetes and CKD, assessing cognitive levels based on estimated glomerular filtration rate (eGFR).

**Methods:**

We conducted a study involving consecutive frail older patients with hypertension recruited from March 2021 to March 2023 at the ASL (local health unit of the Italian Ministry of Health) of Avellino, Italy, followed up after three months. Inclusion criteria comprised age over 65 years, prior diagnosis of hypertension without secondary causes, prediabetes, frailty status, Montreal Cognitive Assessment (MoCA) score < 26, and CKD with eGFR > 15 ml/min.

**Results:**

237 patients completed the study. We examined the association between albuminuria and MoCA Score, revealing a significant inverse correlation (r: 0.8846; *p* < 0.0001). Subsequently, we compared MoCA Score based on eGFR, observing a significant difference (*p* < 0.0001). These findings were further supported by a multivariable regression analysis, with albuminuria as the dependent variable.

**Conclusions:**

Our study represents the pioneering effort to establish a significant correlation between albuminuria and eGFR with cognitive function in frail hypertensive older adults afflicted with prediabetes and CKD.

## Background

Hypertension and chronic kidney disease (CKD) pose significant public health challenges, sharing intertwined pathophysiological mechanisms [1–3]. Elevated blood pressure contributes to kidney function decline, while CKD exacerbates hypertension [1, 4, 5]. Both conditions are associated with aging and frailty, contributing to adverse outcomes such as cognitive and physical impairments, driving the onset of disability, hospitalization, and death [6–12]. Frailty is an increasingly prevalent condition in older adults and is defined by the presence of at least three of five Fried criteria [13]; on the other hand, pre-frailty is a condition that precedes frailty defined by the presence of one or two Fried criteria [13]. Prediabetes is recognized as a precursor to diabetes, diagnosed with HbA1c values between 5.7 and 6.4% [14, 15], and is often accompanied by cardiovascular comorbidities, such as hypertension, thereby elevating the risk of pre-frailty and frailty [16, 17].

Albuminuria serves as a hallmark of kidney disease, subclinical cardiovascular issues, and organ damage in hypertension [18–27] amplifying the risk of pre-frailty, frailty, and cognitive decline in older adults [28–33]. Moreover, albuminuria may underlie endothelial dysfunction, prevalent in both hypertension and CKD [33–37]. Hence, in the present study we explored the association between albuminuria and cognitive impairment in frail older adults with prediabetes and CKD, assessing cognitive levels based on estimated glomerular filtration rate (eGFR).

## Methods

We designed a study involving consecutive frail older patients with hypertension and prediabetes recruited from March 2021 to March 2023 at the ASL (local health unit of the Italian Ministry of Health) of Avellino, Italy. The study was named “CARYATID”: **C**ognitive perform**A**nce f**R**ail h**Y**pertensive **A**dul**T**s w**I**th ck**D**. Inclusion criteria comprised age over 65 years, prior diagnosis of hypertension without secondary causes, prediabetes, frailty status, Montreal Cognitive Assessment (MoCA) score < 26 [38], and CKD with eGFR > 15 ml/min. Albuminuria was defined using a cutoff of 30 mg/dl in 24-hour urine [39]. Informed consent was obtained from each patient or their legal representative, and the research adhered to the principles outlined in the 1975 Declaration of Helsinki and its subsequent revisions. The Institutional Review Board of Campania Nord approved the protocol.

### Global cognitive function evaluation

Global cognitive function has been assessed via MoCA test. This cognitive test covers many cognitive skills, and scores range from 0 to 30 and cognitive impairment is defined from values < 26 [38, 40].

### Frailty assessment

A physical frailty assessment was performed following the Fried Criteria at baseline; a diagnosis of frailty status was performed with at least three points out of five criteria: low physical activity level, weight loss, exhaustion, weakness, slowness [13, 41].

### Statistical analysis

Data are presented as mean ± SD or percentages. We correlated albuminuria with MoCA Score and gait speed. Afterwards, we compared global cognitive performance whether the eGFR was < 60 or > 60. The correlation between MoCA score and albuminuria was evaluated using the Spearman’s rank test. We also performed a multivariable regression analysis with albuminuria as the dependent variable adjusting for potential confounding factors. All calculations were computed using the SPSS 26 software.

## Results

**Fig. 1:**
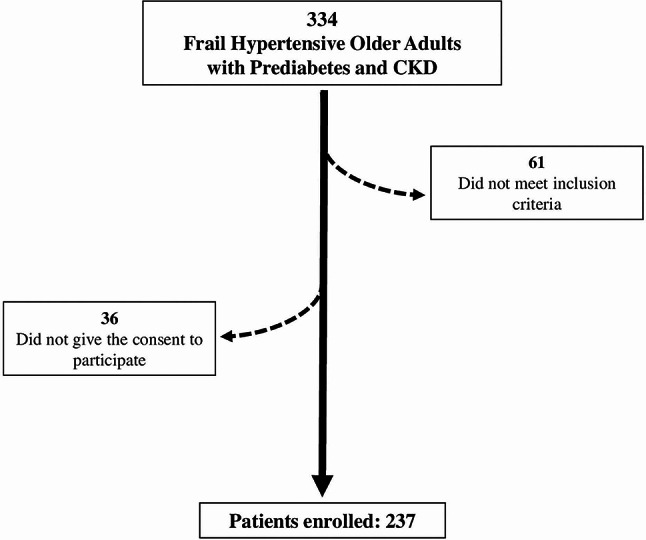
Flow chart of the study

We assessed 334 frail hypertensive elders with prediabetes and CKD. Among them, 61 did not meet the inclusion criteria and 36 declined to participate, leaving 237 patients in our database; the flow-chart of the study is depicted in Fig. 1. The baseline characteristics of our population are shown in Table 1.

**Table 1:** Baseline clinical characteristics of our population

N	237
Mean age (years)	78.4 ± 8.2
BMI (kg/m^2^)	27.6 ± 4.4
SBP (mmHg)	126.2 ± 10.9
DBP (mmHg)	75.3 ± 10.7
Heart rate (bpm)	79.0 ± 12.1
Albuminuria	249.3 ± 215.5
MoCA	19.3 ± 3.7
Fasting Plasma Glucose (mg/dL)	112.3 ± 8.1
HbA1c (mMol/L)	5.9 ± 0.2
Creatinine	1.3 ± 0.2
Dyslipidemia	108 (45.6)
COPD	83 (35.0)
Osteoarthritis	106 (44.7)

Data are means ± SD. BMI: body mass index; SBP: systolic blood pressure; DBP: diastolic blood pressure; MoCA: montreal cognitive assessment; eGFR: estimated glomerular filtration rate; COPD: chronic obstructive pulmonary disease

**Fig. 2:**
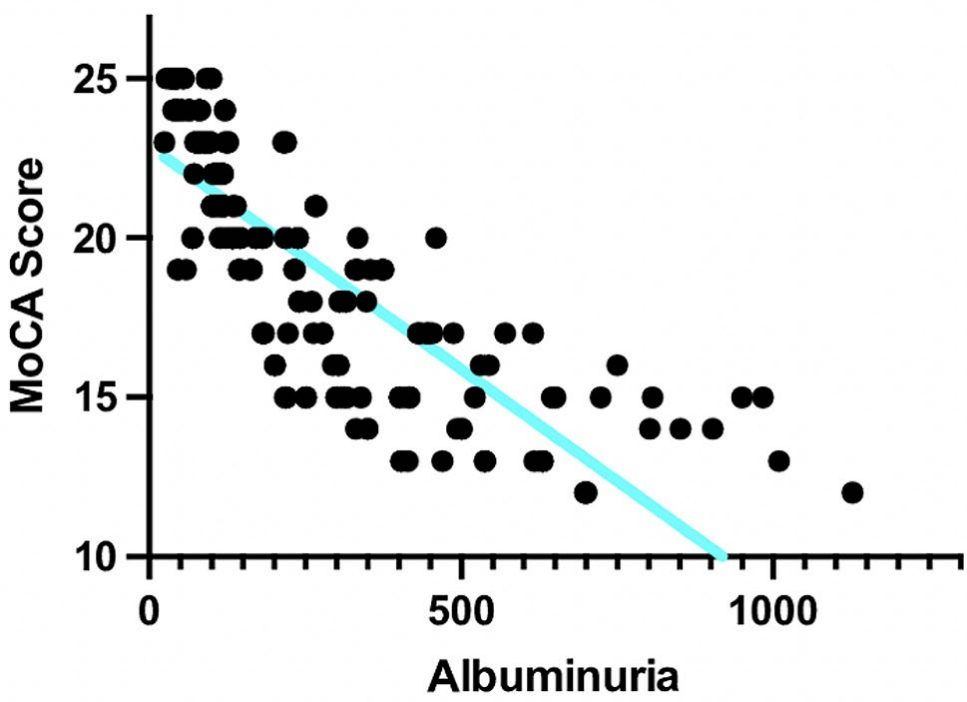
Inverse correlation between MoCA Score and albuminuria in frail hypertensive older adults with prediabetes and CKD (r: 0.8846; 95%CI: -0.9114 to -0.8505; *p* < 0.0001)

First, we examined the correlation between albuminuria and MoCA Score, revealing a statistically significant result (r: 0.8846; 95%CI: -0.9114 to -0.8505; *p* < 0.0001; Fig. 2).

**Fig. 3:**
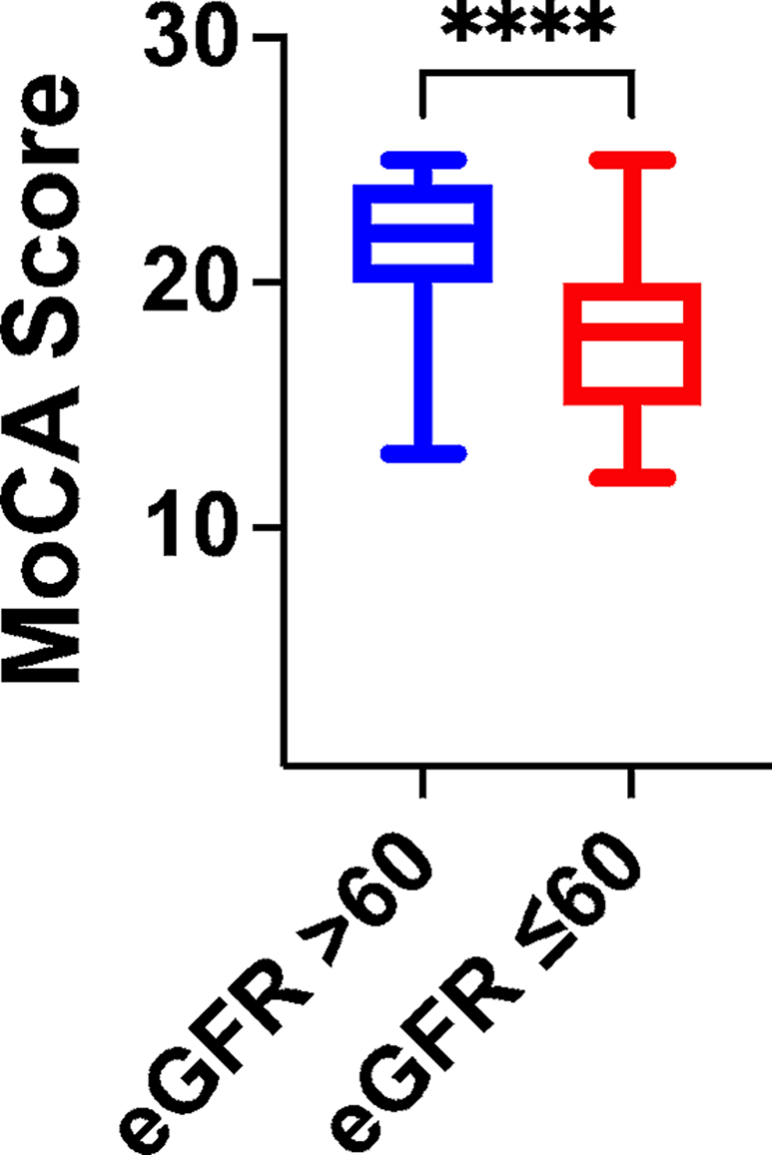
Assessment of MoCA score in our population divided by eGFR value (****: *p* < 0.0001)

Subsequently, we sought to assess if there was any difference in the values of MoCA Score when subdividing our population in two groups, based on their eGFR and we observed that the MoCA score was significantly reduced in patients with eGFR ≤ 60 vs. patients with eGFR > 60 (*p* < 0.0001; Fig. 3).

These findings were further supported by a multivariable regression analysis, with albuminuria as the dependent variable, as shown in Table 2.

**Table 2:** Multivariable regression analysis with albuminuria as the dependent variable

	B	SD	Beta	t	p	95% Confidence interval for B	
						Lower bound	Upper bound
MoCA	-37,209	2,900	-,648	-12,829	,000	-42,923	-31,494
Age	6,107	1,313	,233	4,650	,000	3,519	8,694
Dyslipidemia	-1,885	16,400	-,004	-,115	,909	-34,198	30,428
COPD	19,864	16,825	,044	1,181	,239	-13,286	53,014
Osteoarthritis	11,996	16,183	,028	,741	,459	-19,890	43,881

COPD: Chronic Obstructive Pulmonary disease; MoCA: montreal cognitive assessment

## Discussion

We conducted a comprehensive analysis to investigate the relationship between albuminuria, a marker of kidney damage, and the MoCA Score, a measure of cognitive function. Our results revealed a robust and statistically significant correlation between albuminuria and MoCA Score, indicating that higher levels of albumin in the urine were associated with lower cognitive performance. Additionally, we sought to explore the impact of eGFR on MoCA Score and found a significant difference in cognitive function based on eGFR levels, further highlighting the importance of kidney function in cognitive health. To validate these findings, we performed a multivariable regression analysis, with albuminuria as the dependent variable, which confirmed the significant association between albuminuria and cognitive impairment even after adjusting for potential confounding factors. Intriguingly, age was significant (*p* < 0.001), confirming the importance of aging in cognitive decline and frailty [42, 43].

Our observations are consistent with previous reports indicating that CKD often complicates hypertension [2, 24, 44], and that, likewise, prediabetes exacerbates kidney dysfunction [45–48]. Besides, albuminuria serves as a common marker of organ damage and endothelial dysfunction [49, 50] and the relationship between cognitive dysfunction/impairment and albuminuria is widely accepted [51, 52].

Thus, the relationship between CKD and hypertension is bidirectional and complex; hypertension can contribute to the development and progression of CKD by placing increased pressure on the kidneys over time, leading to damage to the blood vessels in the kidneys and impairing their ability to filter waste products and excess fluids from the blood effectively; conversely, CKD can also exacerbate hypertension by causing changes in the body’s fluid and electrolyte balance, leading to elevated blood pressure levels [53, 54]. Equally important, prediabetes is known to exacerbate kidney dysfunction [55]. In this context, cognitive impairment represents a prevalent complication among these individuals, particularly in frail patients who face an increased risk of cognitive decline, with or without dementia [56, 57]. Consequently, albuminuria may serve as a prognostic indicator for poorer cognitive performance within this subgroup [58–60]. Notably, in our study, eGFR levels significantly influence overall cognitive function, with a cutoff of 60 indicating lower MoCA Scores (*p* < 0.0001).

Our clinical study does not allow to determine the exact mechanisms underlying the association between albuminuria and cognitive dysfunction. A possibility is that endothelial dysfunction could play a pivotal role in precipitating adverse outcomes in hypertension and prediabetes, contributing to functional decline in frail older adults. Indeed, albuminuria may denote endothelial dysfunction [35, 61, 62], a condition highly prevalent in both hypertension and CKD [33, 34, 36, 37, 63, 64]. We also speculate that CKD patients may have vascular brain damage, exacerbating cognitive decline. This hypothesis may be confirmed by our Fig. 3 in which lower levels of eGFR are suggesting a worst global cognitive function. Indeed, vascular risk factors are known to contribute to both vascular and Alzheimer’s dementia [65, 66]. Limitations of our study include not having adjusted for current medications and not having obtained from each patients the socio-economic status, which might have influenced cognitive function [67–70].

Taken together, our results underscore the intricate interplay between kidney function and cognitive health, indicating that early detection and management of kidney damage may play a crucial role in preserving cognitive function. Thus, managing blood pressure and glycemia becomes paramount in preventing organ damage and associated complications. While our findings are significant, further investigations with larger sample sizes are necessary to validate our results.

## Conclusions

We demonstrated a significant correlation between albuminuria and cognitive dysfunction, also showing that the MoCA score is significantly reduced in patients with eGFR ≤ 60 compared to patients with eGFR > 60, a finding corroborated by a multivariable regression analysis, adjusting for potential confounders. Our study represents the pioneering effort to establish a significant correlation between albuminuria and eGFR with cognitive function in frail hypertensive older adults afflicted with prediabetes and CKD.

## Data Availability

The data that support the findings of this study are available from the last author upon reasonable request.
