# Correction to: Frail hypertensive older adults with prediabetes and chronic kidney disease: insights on organ damage and cognitive performance - preliminary results from the CARYATID study

**DOI:** 10.1186/s12933-024-02262-7

**Published:** 2024-05-29

**Authors:** Gaetano Santulli, Valeria Visco, Michele Ciccarelli, Mario Nicola Vittorio Ferrante, Piero De Masi, Antonella Pansini, Nicola Virtuoso, Armando Pirone, Germano Guerra, Veronica Verri, Gaetano Macina, Alessandro Taurino, Klara Komici, Pasquale Mone

**Affiliations:** 1 Department of Medicine, Division of Cardiology, Wilf Family Cardiovascular Research Institute, Einstein? Mount Sinai Diabetes Research Center (ES-DRC), Einstein Institute for Aging Research, Albert Einstein College of Medicine, New York, NY USA; 2 Department of Molecular Pharmacology, Einstein Institute for Neuroimmunology and Inflammation (INI), Fleischer Institute for Diabetes and Metabolism (FIDAM), Albert Einstein College of Medicine, New York, NY USA; 3 Department of Advanced Biomedical Sciences, University of Naples, ?Federico II?, Fisciano, Italy; 4 International Translational Research and Medical Education (ITME) Consortium, Academic Research Unit, Naples, Italy; 5 Department of Medicine, Surgery and Dentistry, University of Salerno, Baronissi, Italy; 6 ASL Avellino, Avellino, Italy; 7 Cardiology Unit, University Hospital ?San Giovanni di Dio e Ruggi d?Aragona?, Salerno, Italy; 8 Department of Medicine and Health Sciences ?Vincenzo Tiberio?, University of Molise, Campobasso, Italy; 9 University of Bari, Bari, Italy; 10 Casa di Cura ?Montevergine?, Mercogliano, Avellino Italy

**Correction to: Cardiovascular Diabetology (2024) 23:125**

10.1186/s12933-024-02218-x

Following publication of the original article [1], the corresponding author names were incorrectly published by the typesetter during correction process. The author ?Gaetano Santulli? was inadvertently denoted as the corresponding author but the corresponding authorship should have been to both the authors ?Gaetano Santulli and Pasquale Mone? as per accepted manuscript. We apologize for the error and this has been corrected with this erratum.

Also, the affiliations for Dr. Pasquale Mone has been corrected who is affiliated with ASL Avellino instead of Salerno University.

The original article has been corrected.
